# Lichen-Derived Depsides and Depsidones Modulate the Nrf2, NF-κB and STAT3 Signaling Pathways in Colorectal Cancer Cells

**DOI:** 10.3390/molecules26164787

**Published:** 2021-08-07

**Authors:** Katarzyna Papierska, Violetta Krajka-Kuźniak, Jarosław Paluszczak, Robert Kleszcz, Marcin Skalski, Elżbieta Studzińska-Sroka, Wanda Baer-Dubowska

**Affiliations:** 1 Department of Pharmaceutical Biochemistry, Poznan University of Medical Sciences, 4 Święcicki Str., 60-781 Poznań, Poland; kpapierska@ump.edu.pl (K.P.); paluszcz@ump.edu.pl (J.P.); kleszcz@ump.edu.pl (R.K.); marcinskalski1993@gmail.com (M.S.); baerw@ump.edu.pl (W.B.-D.); 2 Department of Pharmacognosy, Poznan University of Medical Sciences, 4 Święcicki Str., 60-781 Poznań, Poland; ela_studzinska@op.pl

**Keywords:** colorectal cancer, lichens, NF-κB, Nrf2, STAT3

## Abstract

The study aimed to evaluate the possible modulation of Nrf2, NF-ĸB and STAT3 signaling pathways in the colorectal cancer (CRC) cells line DLD-1 and HCT116 by secondary metabolites of lichens. An attempt was made to indicate the most promising targets in these signaling pathways. Attention was also paid to the effects of the compounds tested on CRC cells using anakoinosis—that is, simultaneous analysis of several signaling pathways. The effects of the tested natural compounds on the activity of selected transcriptional factors related to CRC were analyzed by Western blot and RT-PCR assays. The highest activity against CRC cells was shown by physodic and salazinic acids from the studied secondary metabolites of lichens. As a result, an increase in the activation of transcription factor Nrf2 and the expression of its selected target genes was observed. Physodic and salazinic acids induced the opposite effect in relation to the NF-κB and STAT3 pathways. These results confirmed our earlier observations that lichen-derived compounds have the ability to modulate signaling pathway networks. While caperatic acid affected Wnt/β-catenin to the most extent, salazinic acid was the most potent modulator of Nrf2, NF-κB and STAT3 pathways. Physodic acid seemed to affect all the investigated pathways.

## 1. Introduction

Lichens are the symbionts of nutritionally specialized fungi (mycobiont) that derive fixed carbon from algae and/or cyanobacteria (photobionts) and are present in ecosystems worldwide [1]. Lichens produce numerous secondary metabolites and, currently, over 1000 lichen substances are known [2].

Secondary metabolites present in lichens comprise aliphatic, cycloaliphatic, aromatic and terpenic compounds, which are unique with respect to those of higher plants and show interesting biological and pharmacological activities [3]. However, only a few of these compounds have been assessed for their potential chemopreventive and/or therapeutic activities toward cancer. In this regard, a depside, atranorin, and a depsidone, physodic acid, both synthesized by lichens in the acetyl-polymalonyl pathways, have been shown to display cytotoxic effects in breast [4] and melanoma cancer cells [3], activating an apoptotic process in the latter cells. The acetone extract from *H. physodes*, the major source of physodic acid, demonstrated significant antioxidant properties [4]. On the other hand, atranorin was not cytotoxic and protected SH-SY5Y neuroblastoma cells against H_2_O_2_-induced cell viability impairment. It was suggested that atranorin may act as a pro-oxidant or antioxidant agent depending on the radical [5].

The reduction of cell proliferation and the induction of apoptosis by lichen compounds was also shown in colorectal cancer (CRC) cells [6,7]. Moreover, a decrease in tumor burden resulting from treatment with lichen extracts was observed in animal CRC models [8]. It is generally accepted that aberrations in Wnt/β-catenin signaling lead to the initiation and progression of CRC [9].

Our earlier studies showed that lichen-derived depsides (atranorin, lecanoric acid, squamatic acid), depsidones (physodic acid, salazinic acid) and a polycarboxylic fatty acid (caperatic acid) affect the Wnt/β-catenin signaling in HCT116 and DLD-1 colorectal cancer cell lines [10]. Caperatic acid and physodic acid were shown to be the most effective modulators of β-catenin-dependent transcription, reducing the expression of its target genes: *Axin2*, *survivin* and *MMP7*. DLD-1 cells were more resistant to the inhibitory effects of lichen chemicals. Besides Wnt/β-catenin pathway activation, aberrations in the homeostatic function of Nrf2, NF-κB and STAT3 signaling pathways are also considered important causative factors implicated in colon cancer development [11]. Hence, intervention in these pathways by compounds such as secondary metabolites of lichens may provide a new approach for colon cancer prevention and/or treatment.

As a consequence of their activation, chronic inflammation promotes cell division and creates an environment that stimulates the formation and growth of cancer cells [12]. The sites of the interconnection of the NF-κB, Nrf2 and STAT3 signaling pathways and aberrations in their activation mechanisms make it possible to use them as targets in cancer cells for potential chemotherapeutic compounds. This approach fits with a new trend called “anakoinosis”, the name of which derives from the ancient Greek term for “communication.” Induction of signaling in tumor cells aims to establish novel communication behavior between tumor tissues or between tumor tissue and the host by remodulating gene expression. The concept of anakoinosis is an alternative to conventional chemotherapy, which is usually based on a single target or focused on a single area of the tumor. Unlike standard therapies, treatment protocols based on anakoinosis are less toxic and less likely to lead to drug resistance.

Nrf2, a bZip transcription factor and a member of the Cap‘n’Colar family of regulatory proteins, is sequestered in an inactive form in the cytoplasm by Keap 1 inhibitory protein. Nrf2 is activated by two mechanisms that include the stabilization of Nrf2 via Keap1 cysteine thiol modification and phosphorylation of Nrf2 by upstream kinases. Oxidants such as reactive oxygen species and reactive nitrogen species, but also reactive electrophilic metabolites and some chemopreventive agents, react with redox reactive cysteines in Keap1, disrupting the interaction between Nrf2 and Keap1, hence allowing the translocation of Nrf2 into the cell nucleus. Binding to the ARE sequence results in increased expression of antioxidant and cytoprotective enzymes, including superoxide dismutase (SOD), catalase (CAT), glutathione peroxidase (GPx) and glutathione S-transferases (GSTs) [13,14]. Interestingly, glycogen synthase kinase-3β (GSK-3β) and Fyn tyrosine kinase have been shown to attenuate the transcriptional activity of Nrf2 [15]. In addition, GSK-3β has been reported to be necessary for the full transcriptional activity of NF-κB, demonstrating that GSK-3β selectively supports the expression of a subset of genes activated by NF-κB-dependent proliferative signals [16]. These observations indicate the existence of cross-talk between these two pathways.

In unstimulated cells, NF-κB is sequestered in the cytoplasm through a tight association with its inhibitor, the IκBα protein. The activation of NF-κB occurs through the site-specific phosphorylation of IκBα, leading to the degradation of the inhibitory subunit, which allows free heterodimers to migrate to the cell nucleus. In most cell types, NF-κB activity is mediated by two major complexes: p50/p65 (RelA), which acts as a transcriptional activator, and p50/p50, which functions as a transcriptional repressor [17]. The major target proteins controlled by NF-κB-driven transactivation are cyclooxygenase-2 (COX-2) and inducible nitric oxide synthase (iNOS).

NF-κB and STAT3 are activated in response to various stimuli, including stresses and cytokines, although they are regulated by different signaling mechanisms. STAT3 regulates the expression of various genes in response to cellular stimuli and thus plays a key role in cell growth and apoptosis. The activation and interaction between STAT3 and NF-κB play vital roles in the control of the communication between cancer cells and inflammatory cells. Moreover, STAT3 and its target gene products, including Bcl-xl, have been found to be overexpressed in colon cancer cells. For this reason, the reduced expression of these genes under the influence of lichen metabolites could constitute their chemopreventive or therapeutic target point [18].

**Figure 1:**
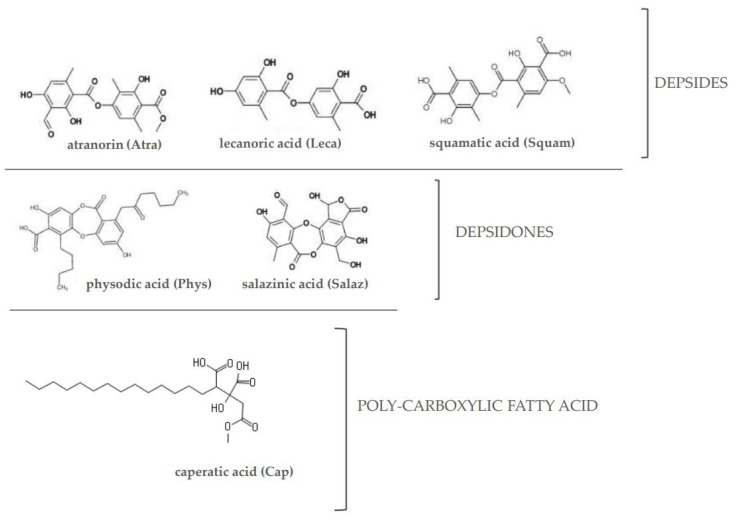
The chemical structure of the investigated lichen-derived compounds.

Taking into consideration the association between these three signaling pathways and their involvement in CRC development, the aim of this study was to evaluate the effects of a poly-carboxylic fatty acid (caperatic acid (Cap)), depsides (atranorin (Atra), lecanoric acid (Leca) and squamatic acid (Squam)) and depsidones (physodic acid (Phys) and salazinic acid (Salaz)) (Figure 1) on the Nrf2, NF-κB and STAT3 transcription factors and the level of expression of their target genes in the same cell lines.

## 2. Results

### 2.1. The Effect of Lichen Secondary Metabolites on the Activation of the Nrf2

**Figure 2:**
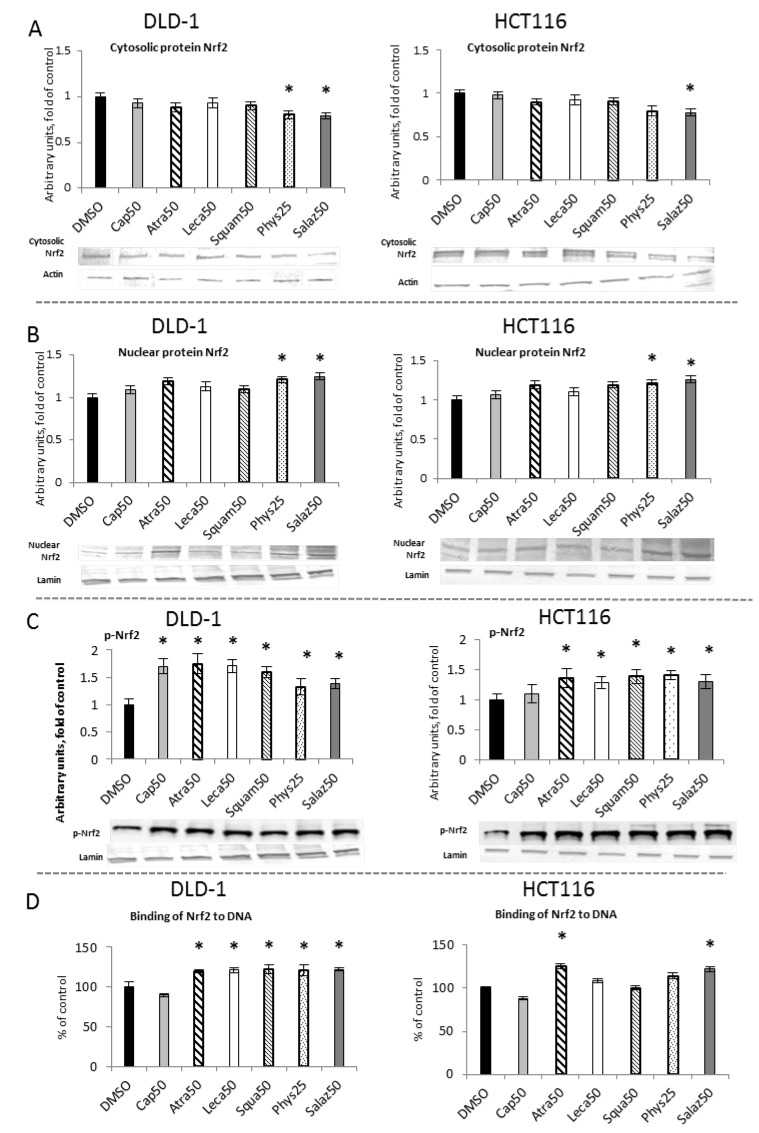
The effect of lichen-derived compounds on Nrf2 activation in DLD-1 and HCT116 cells. (**A**) The level of Nrf2 protein in the cytosolic fraction. (**B**) The level of Nrf2 protein in the nuclear fraction. (**C**) The level of p-Nrf2 protein in the nuclear fraction. Representative Western immunoblots are presented under the graphs (**A**–**C**). Results (means ± SEM from three separate experiments) are presented as a fold change to control after normalization against the level of actin or lamin. (**D**) The level of Nrf2 binding to DNA. Results are presented as the means ± SEM from three separate experiments percentage of control). Asterisks (*) denote statistically significant changes from the control group (*p* ≤ 0.05). **DMSO**, vehicle control; **Cap50**, caperatic acid (50 µM); **Atra50**, atranorin (50 µM); **Leca50**, lecanoric acid (50 µM); **Squam50**, squamatic acid (50 µM); **Phys25**, physodic acid (25 µM); **Salaz50**, salazinic acid (50 µM).

The activation of Nrf2 requires its translocation from the cytosol to the nucleus its release from the complex with the inhibitory protein Keap1. As shown in Figure 2A,B, the levels of Nrf2 in the cytosolic fractions in cells of both lines were lowered by Phys and Salaz, leading to increases in the levels of Nrf2 protein in the nuclei of the cells by about 21% and 25%, respectively.

The amount of Nrf2 bound to the ARE sequence in complex with the DNA was measured to confirm the activation of Nrf2 by the metabolites of the lichens. As shown in Figure 2C, in both cell lines, Atra and Salaz increased the binding level by 20% to 23% compared to the control group. In DLD-1 cells, a similar effect was also observed after treatment with Leca, Squam and Phys.

The level of phosphorylated Nrf2 (p-Nrf2) was measured in the nuclear fraction of the tested cells (Figure 2D). In DLD-1 cells, a significant increase in p-Nrf2 was found under the influence of all tested compounds, and the greatest increase was found for the cases of Atra (75%), Cap (70%) and Leca (71%). These compounds had a significantly smaller effect on the level of p-Nrf2 in HCT116 cells, although an increase from 25% to 41% was also observed in these cells.

### 2.2. The Effect of Lichen Secondary Metabolites on the Expression of Nrf2-Dependent Genes

**Figure 3:**
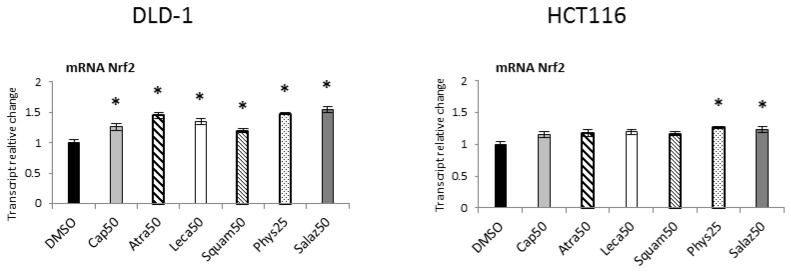
The effect of lichen-derived compounds on the expression of *Nrf2* in DLD-1 and HCT116 cells. The values (fold of control) are presented as the means ± SEM from three separate experiments. Asterisks (*) denote statistically significant changes from the control group (*p* ≤ 0.05). **DMSO**, vehicle control; **Cap50**, caperatic acid (50 µM); **Atra50**, atranorin (50 µM); **Leca50**, lecanoric acid (50 µM); **Squam50**, squamatic acid (50 µM); **Phys25**, physodic acid (25 µM); **Salaz50**, salazinic acid (50 µM).

The effect of Nrf2 activation was the expression of target genes encoding antioxidant enzymes and proteins, as well as phase II enzymes of xenobiotic metabolism and the *Nrf2* gene. As shown in Figure 3, all the lichen metabolites tested increased the transcript level of the *Nrf2* gene, especially in the DLD-1 cell line. In HCT116 cells, the increase in the mRNA level of this gene was lower but similar to that of DLD-1 cells, and Phys and Salaz were the most prominent inducers.

**Figure 4:**
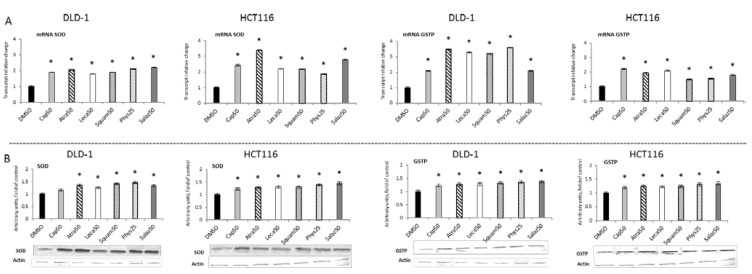
The effect of lichen-derived compounds on the expression of selected Nrf2 target genes: SOD and GSTP in DLD-1 and HCT116 cells. (**A**) Levels of *SOD* and *GSTP* transcripts. The values (fold of control) are presented as the means ± SEM from three separate experiments. (**B**) Level of SOD and GSTP proteins. Representative Western immunoblots are presented under the graphs. Results (means ± SEM from three separate experiments) are presented as a fold change to control after normalization against the level of actin. Asterisks (*) denote statistically significant changes from the control group (*p* ≤ 0.05). **DMSO**, vehicle control; **Cap50**, caperatic acid (50 µM); **Atra50**, atranorin (50 µM); **Leca50**, lecanoric acid (50 µM); **Squam50**, squamatic acid (50 µM); **Phys25**, physodic acid (25 µM); **Salaz50**, salazinic acid (50 µM).

**Figure 5:**
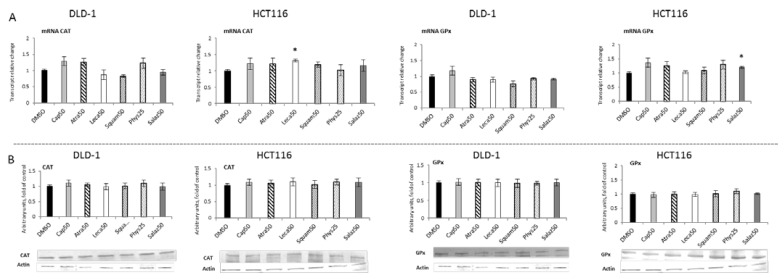
The effect of lichen-derived compounds on the expression of selected Nrf2 target genes: CAT and GPx in DLD-1 and HCT116 cells. (**A**) Levels of *CAT* and *GPx* transcripts. The values (fold of control) are presented as the means ± SEM from three separate experiments. (**B**) Levels of CAT and GPx proteins. Representative Western immunoblots are presented under the graphs. Results (means ± SEM from three separate experiments) are presented as a fold change to control after normalization against the level of actin. Asterisks (*) denote statistically significant changes from the control group (*p* ≤ 0.05). **DMSO**, vehicle control; **Cap50**, caperatic acid (50 µM); **Atra50**, atranorin (50 µM); **Leca50**, lecanoric acid (50 µM); **Squam50**, squamatic acid (50 µM); **Phys25**, physodic acid (25 µM); **Salaz50**, salazinic acid (50 µM).

Figure 4 and Figure 5 present the results of the analysis of the *SOD, GSTP, CAT* and *GPx* gene expressions. All the tested compounds increased *GSTP* and *SOD* mRNA levels in both DLD-1 and HCT116 cells. Among the analyzed compounds, Atra turned out to be the most potent inducer of *SOD* transcription in the HCT116 cell line, increasing its transcript level by about 200% compared to the control group. In turn, the level of *GSTP* mRNA was most strongly increased by Phys—about 260% (Figure 4A) in the DLD-1 cells. Furthermore, the cytosolic contents of SOD, GSTP, CAT and GPx were assessed in order to verify whether the changes in the transcript level were reflected in their protein levels. The results are shown in Figure 4 and Figure 5. Basically, the changes in the protein levels of GSTP and SOD in the HCT116 and DLD-1 cells confirmed the increased expression of the genes encoding these enzymes. However, the most significant increase was found after treatment with Phys and Salaz (from 25% to 45%) in both HCT116 and DLD-1 cell lines.

### 2.3. The Effect of Lichen Secondary Metabolites on the Activation of the NF-κB

**Figure 6:**
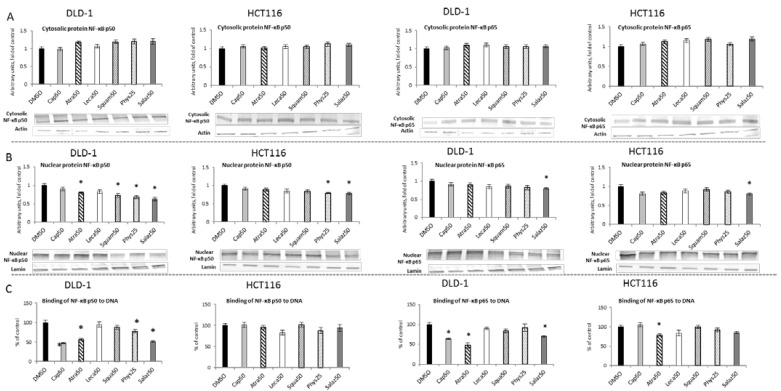
The effect of lichen-derived compounds on NF-κB activation in DLD-1 and HCT116 cells. (**A**) The levels of NF-κB p50 and p65 proteins in the cytosolic fraction. (**B**) The levels of NF-κB p50 and p65 proteins in the nuclear fraction. Representative Western immunoblots are presented under the graphs (**A**,**B**). Results (means ± SEM from three separate experiments) are presented as a fold change to control after normalization against the level of actin or lamin. (**C**) The levels of NF-κB p50 and p65 binding to DNA. Results are presented as the means ± SEM from three separate experiments percentage of control). Asterisks (*) denote statistically significant changes from the control group (*p* ≤ 0.05). **DMSO**, vehicle control; **Cap50**, caperatic acid (50 µM); **Atra50**, atranorin (50 µM); **Leca50**, lecanoric acid (50 µM); **Squam50**, squamatic acid (50 µM); **Phys25**, physodic acid (25 µM); **Salaz50**, salazinic acid (50 µM).

Activation of NF-κB as a result of degradation of the cytosolic inhibitory protein IκB leads to translocation of active dimers into the nucleus and binding to the target sequence in DNA. Figure 6 presents the effect of lichen-derived compounds on the cytosolic and nuclear levels of NF-κB in DLD-1 and HCT116 cells. Under the influence of Salaz and Phys, the level of the p50 subunit protein in the nuclear fraction decreased in the cells of both of the tested cell lines (Figure 6B). In DLD-1 cells, this effect was much more pronounced, with a decrease of 20% to 37% under Atra and Squam’s influence compared to the control group. In the case of the p65 subunit, the level of its protein was reduced by 20% in the nuclear fraction only under the influence of Salaz in the cells of both tested cell lines.

A significant reduction in the binding of both the p50 and p65 subunits (Figure 6C) to DNA in DLD-1 cells was demonstrated by the action of Cap, Atra and Salaz. Additionally, Phys decreased the level of p50 binding to DNA in DLD-1 cells by 21%. On the other hand, in HCT116 cells, a significant effect of lowering the p65 binding level (by 21% compared to the control group) was only observed under the influence of Atra.

### 2.4. The Effect of Lichen Secondary Metabolites on the Expression of NF-κB-Dependent Genes

**Figure 7:**
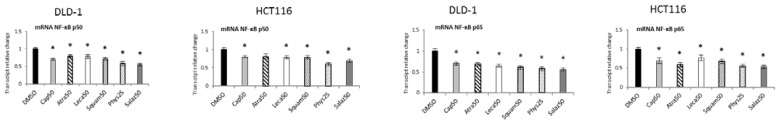
The effect of lichen-derived compounds on the expression of NF-κB p50 and p65 in DLD-1 and HCT116 cells. The values (fold of control) are presented as the means ± SEM from three separate experiments. Asterisks (*) denote statistically significant changes from the control group (*p* ≤ 0.05). **DMSO**, vehicle control; **Cap50**, caperatic acid (50 µM); **Atra50**, atranorin (50 µM); **Leca50**, lecanoric acid (50 µM); **Squam50**, squamatic acid (50 µM); **Phys25**, physodic acid (25 µM); **Salaz50**, salazinic acid (50 µM).

Figure 7 shows the transcript levels of both the tested NF-κB subunits in DLD-1 and HCT116 cells after treatment with active lichen secondary metabolites. The transcript levels of the NF-ĸB p50 and p65 subunits were lowered after treatment with all tested compounds in both DLD-1 and HCT116 cells. Phys and Salaz turned out to be the most potent inhibitors of NF-ĸB p50 and NF-ĸB p65 transcription, leading to reductions in their mRNA levels of 32% and 45%, respectively, compared to the control cells.

**Figure 8:**
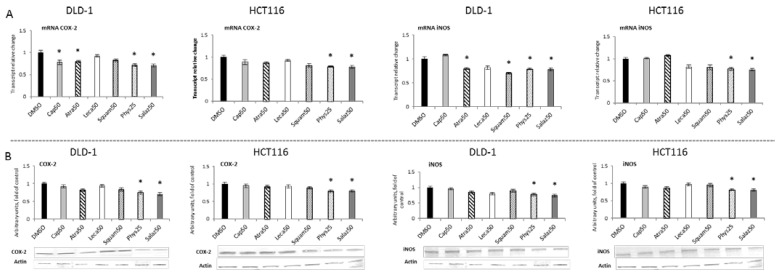
The effect of lichen-derived compounds on the expression of selected NF-κB target genes: COX-2 and *iNOS* in DLD-1 and HCT116 cells. (**A**) Levels of COX-2 and *iNOS* transcripts. The values (fold of control) are presented as the means ± SEM from three separate experiments. (**B**) Levels of COX-2 and *iNOS* proteins. Representative Western immunoblots are presented under the graphs. Results (means ± SEM from three separate experiments) are presented as a fold change to control after normalization against the level of actin. Asterisks (*) denote statistically significant changes from the control group (*p* ≤ 0.05). **DMSO**, vehicle control; **Cap50**, caperatic acid (50 µM); **Atra50**, atranorin (50 µM); **Leca50**, lecanoric acid (50 µM); **Squam50**, squamatic acid (50 µM); **Phys25**, physodic acid (25 µM); **Salaz50**, salazinic acid (50 µM).

The activation of NF-κB leads to the transcription of several genes, including *COX-2* and *iNOS*, coding for cyclooxygenase-2 and nitric oxide synthase, respectively. As shown in Figure 8A,B, Phys and Salaz significantly reduced COX-2 expression at both transcript and protein levels by 20% to 30% in DLD-1 and HCT116 cells. Moreover, a reduction in COX-2 mRNA was also observed in the DLD-1 line as the effect of treatment with Atra and Cap.

The reduction in the iNOS mRNA level by Phys and Salaz was confirmed for the protein level in the cells of both tested lines (Figure 8). In addition, Atra in the DLD-1 cells and Squam in both the DLD-1 and HCT116 cells significantly reduced the level of the *iNOS* transcript (Figure 8A).

### 2.5. The Effect of Lichen Secondary Metabolites on the Activation of the STAT3

**Figure 9:**
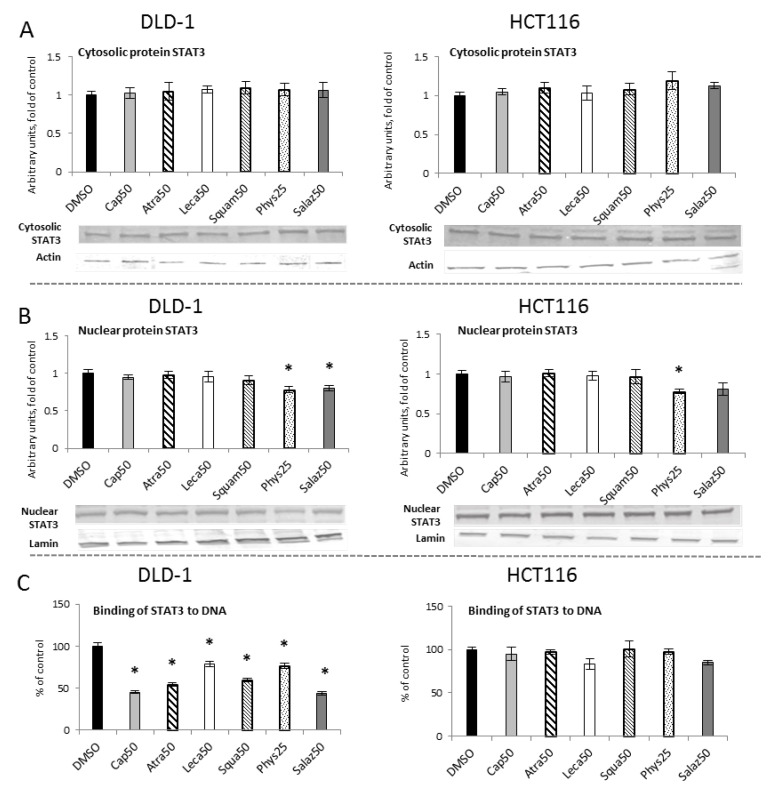
The effect of lichen-derived compounds on STAT3 activation in DLD-1 and HCT116 cells. (**A**) The level of STAT3 protein in the cytosolic fraction. (**B**) The level of STAT3 protein in the nuclear fraction. Representative Western immunoblots are presented under the graphs (**A**,**B**). Results (means ± SEM from three separate experiments) are presented as a fold change to control after normalization against the level of actin or lamin. (**C**) The level of STAT3 binding to DNA. Results are presented as the means ± SEM from three separate experiments percentage of control). Asterisks (*) denote statistically significant changes from the control group (*p* ≤ 0.05). **DMSO**, vehicle control; **Cap50**, caperatic acid (50 µM); **Atra50**, atranorin (50 µM); **Leca50**, lecanoric acid (50 µM); **Squam50**, squamatic acid (50 µM); **Phys25**, physodic acid (25 µM); **Salaz50**, salazinic acid (50 µM).

The level of cytosolic STAT3 protein showed no changes (Figure 9A). However, Western blot analysis of the nuclear STAT3 protein level (Figure 9B) showed a reduction in the STAT3 level in both cell lines with Phys (22%) and only in the DLD-1 line with Salaz (20%).

To assess the activation of STAT3 sequence-specific DNA binding, the ELISA immunoenzymatic test was performed (Figure 9C). In comparison to the control group, in DLD-1 cells we observed a reduced level of STAT3 binding to DNA for all of the analyzed lichen secondary metabolites. This reduction was the most marked for Salaz and amounted to 56%.

**Figure 10:**
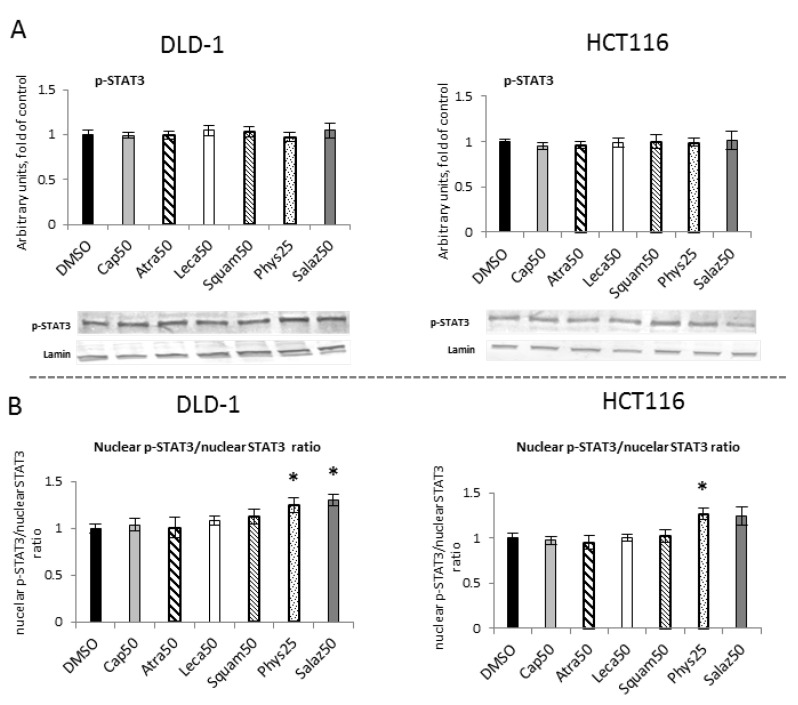
The effect of lichen-derived compounds on the level of p-STAT3 in DLD-1 and HCT116 cells. (**A**) The level of p-STAT3 protein in the nuclear fraction. Representative Western immunoblots are presented under the graphs. Results (means ± SEM from three separate experiments) are presented as a fold change to control after normalization against the level of lamin. (**B**) The ratio of nuclear p-STAT3 and nuclear STAT3 compared with the control group. **DMSO**, vehicle control; **Cap50**, caperatic acid (50 µM); **Atra50**, atranorin (50 µM); **Leca50**, lecanoric acid (50 µM); **Squam50**, squamatic acid (50 µM); **Phys25**, physodic acid (25 µM); **Salaz50**, salazinic acid (50 µM).

To assess the effect of lichen metabolites on the phosphorylation of STAT3 mediated by kinases from the MAP family, the level of phosphorylated STAT3 (p-STAT3) was measured in the nuclear fractions of DLD-1 and HCT116 cells (Figure 10A). However, the p-STAT3 protein level analysis did not show any statistically significant differences under the influence of the tested compounds. The nuclear p-STAT3/STAT3 ratio was calculated and indicated significant changes for Phys and Salaz in DLD-1 cells and for Phys in HCT116 cells (Figure 10B).

### 2.6. The Effect of Lichen Secondary Metabolites on the Expression of STAT3-Dependent Genes

**Figure 11:**
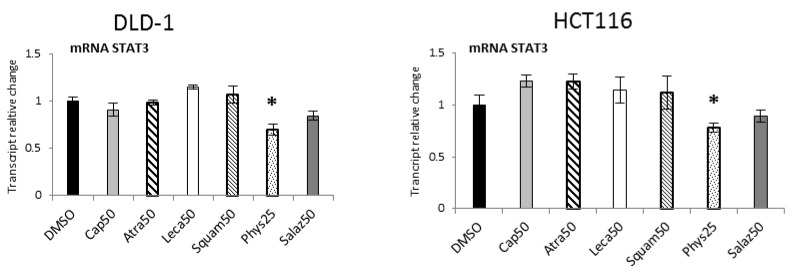
The effect of lichen-derived compounds on the expression of *STAT3* in DLD-1 and HCT116 cells. The values (fold of control) are presented as the means ± SEM from three separate experiments. Asterisks (*) denote statistically significant changes from the control group (*p* ≤ 0.05). **DMSO**, vehicle control; **Cap50**, caperatic acid (50 µM); **Atra50**, atranorin (50 µM); **Leca50**, lecanoric acid (50 µM); **Squam50**, squamatic acid (50 µM); **Phys25**, physodic acid (25 µM); **Salaz50**, salazinic acid (50 µM).

Quantitative analysis of STAT3 expression (Figure 11) showed significant reductions under the influence of Phys in the DLD-1 and HCT116 lines by 30% and 25%, respectively.

**Figure 12:**
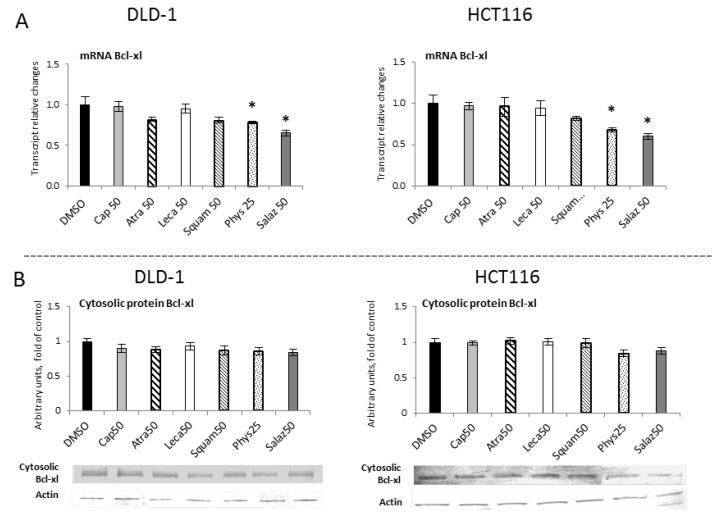
The effect of lichen-derived compounds on the expression of selected STAT3 target gene: Bcl-xl in DLD-1 and HCT116 cells. (**A**) Level of the *Bcl-xl* transcript. The values (fold of control) are presented as the means ± SEM from three separate experiments. (**B**) Level of the Bcl-xl protein. Representative Western immunoblots are presented under the graphs. Results (means ± SEM from three separate experiments) are presented as a fold change to control after normalization against the level of actin. Asterisks (*) denote statistically significant changes from the control group (*p* ≤ 0.05). **DMSO**, vehicle control; **Cap50**, caperatic acid (50 µM); **Atra50**, atranorin (50 µM); **Leca50**, lecanoric acid (50 µM); **Squam50**, squamatic acid (50 µM); **Phys25**, physodic acid (25 µM); **Salaz50**, salazinic acid (50 µM).

The influence of STAT3 on cellular functions can be attributed to some of the gene targets that have been identified for STAT3, such as Bcl-xl. Figure 12 show R-T PCR and Western blot analyses of the Bcl-xl protein, respectively. They show that Salaz was the strongest inhibitor of *Bcl-xl* gene expression at the mRNA level in DLD-1 and HCT116 cells at 35% and 40%, respectively, compared to the control group. Moreover, the *Bcl-xl* transcript was also reduced under the impact of Phys, but the observed effect was much weaker (from 20% to 33%). However, changes in the level of Bcl-xl mRNA were not confirmed at the level of its protein.

## 3. Discussion

Lichens are a known source of unique secondary metabolites, many of which play important ecological roles, including regulating the equilibrium between symbionts. However, only a few of these compounds have been assessed for their anti-cancer effects in in vitro cancer models. Moreover, the mechanisms of the biological activity of lichen secondary metabolites in living cells, including cancer cells, are still poorly known [3].

One potential mechanism of action of anti-cancer agents is influence on signaling pathways, allowing intervention at different levels of signal transduction. The interconnection of signaling pathways such as Wnt/β-catenin, Nrf2-ARE, NF-κB and STAT3 and their aberrations lead to disturbance of cell homeostasis and are the main causative factors involved in the development of colorectal cancer cells. For this reason, simultaneous inhibition of these pathways appears to be an effective way to prevent and/or treat these types of cancer. This applies in particular to compounds of natural origin, including those under investigation in this study—lichen secondary metabolites [19].

Our recent studies showed the ability of physodic acid and caperatic acid to modulate β-catenin-dependent transcription in DLD-1 and HCT116 CRC cell lines [10]. These lichen components reduced the expression of classical β-catenin target genes—*Axin2*, *survivin* and *MMP7*. Recently, it has been found that the Wnt/β-catenin signaling pathway and Nrf2, a major regulator of redox homeostasis, are associated both in vitro and in vivo [19]. The Wnt pathway downstream inhibitory complex of Axin1-GSK-3β proteins may interact with Nrf2, thus holding Nrf2 in the cytoplasm and ultimately leading to its proteasomal degradation [20]. Thus, one might expect that the inhibition of Wnt signaling would inhibit Nrf2 signaling by limiting the entry of Nrf2 into the nucleus. Indeed, such a “double” effect has been described for Wnt inhibitor LGK-974 in hepatocellular carcinoma HepG2 cells. LGK-974 inhibited *c-Myc* gene expression downstream of Wnt3A and also Q*uinone oxidoreductase-1* and *Heme oxygenase-1* Nrf2 target gene expression [21].

The results of the present study proved that lichen components could not inhibit the entry of Nrf2 into the nucleus in DLD-1 and HCT116 CRC cells but, on the contrary, increased the level of Nrf2 in the nuclear fraction and its binding to the ARE regulatory sequences in DNA. Moreover, the evidence for the increased activation of Nrf2 was the increase in the level of its phosphorylated form, p-Nrf2, in the cell nucleus, indicating activation by AMPK [22].

The effect of Nrf2 activation was increased expression of *SOD* and *GSTP* and of *CAT* and *GPx* target genes, including the *Nrf2* gene. Physodic acid and salazinic acid were found to be the most potent inducers of the Nrf2 pathway. The increased expression, especially of the gene encoding the *GSTP* phase II enzyme, was particularly pronounced at the mRNA level but did not find such significant confirmation at the protein level. The lack of correlation between the levels of transcript and protein has been described by many authors. For example, in the studies by Szaefer et al. (2012), an almost six-fold increase in the level of *Nrf2* mRNA was observed under the influence of 3,3-diindolylmethane and only a 1.2-fold increase in the level of protein. Many of the processes occurring between transcription and translation can contribute to this discrepancy. Protein stability is also an important factor, significantly different from mRNA stability [23,24].

Nrf2 plays an essential role in protecting cells from DNA damage and from the deterioration of proteins, carbohydrates and lipids when exposed to oxidative and xenobiotic stress. Thus, impaired function of Nrf2 accounts for many stress-induced pathological disorders, including cancer [25].

Accordingly, the cancer-preventive effects of Nrf2-inducing agents such as oltipraz or sulforaphane were abrogated in Nrf2-deficient mice when Nrf2-dependent gene expression had been shut down [26,27]. In this regard, sulforaphane prevented the development of colon tumors in APC^Min^ mice; however, this effect was abrogated in Nrf2-deficient mice. In addition, mice expressing the abnormal APC protein were shown to be more prone to colon tumor cell formation if the Nrf2 gene was ablated [25,28]. Thus, there is no doubt that Nrf2 can protect against cancer initiation/development, mainly by protecting against genotoxic insults that emerge from exposure to carcinogens and extrinsically or intrinsically generated ROS or electrophiles.

On the other hand, it is also well-established that a great number of tumors frequently exhibit enhanced Nrf2 activity, which may contribute to a malignant phenotype. Nrf2 amplifications range from a frequency of 20%–25% up to 80%–95% in pancreatic, colorectal or prostate cancer [25,29,30,31,32]. Thus, if persistently activated, Nrf2 may become pro-tumorigenic. Studies have also implicated Nrf2 in cancer cell survival and chemo-resistance [30,33]. In this regard, it has been shown that the activation of Nrf2 contributes to the resistance of colorectal adenocarcinoma HT-29 cells to 5-fluorouracil [34]. Therefore, it was postulated that Nrf2 might represent a potential therapeutic target in 5-FU treatment of colon cancer.

More recently, evidence was provided for the elevated expression of Nrf2 in CRC tumor tissue compared with matched normal colonic mucosa. A positive correlation between Nrf2 expression in matched CRC primary and metastatic tissue was also demonstrated. Moreover, it was shown that the inhibition of Nrf2 by brusatol can sensitize CRC HCT116 cells to the cytotoxic effects of irinotecan in vitro and that brusatol can inhibit tumor growth in a syngeneic orthotopic mouse model of CRC. These findings highlight Nrf2 as a potential drug target in the treatment of CRC [29].

Thus, the understanding of the significance of the increased expression and activation of Nrf2 by lichen components in CRC cells requires further studies.

Our study also indicated reductions in the expression and the nuclear protein levels of p50 and p65 NF-κB subunits as a result of treatment with lichen compounds, particularly physodic and salazinic acids. These subunits form a complex that acts as a transcriptional activator. Thus, the reduced levels of these subunits in the nucleus led to diminished expression of the NF-κB target genes COX-2 and *iNOS*.

The cross-talk with NF-κB through GSK-3β involvement plays an important role in shaping the Nrf2 pro-tumorigenic function [35]. Both pathways negatively interfere with each other [36]. Activated NF-κB competes with Nrf2 for CBP coactivators and leads to Nrf2 inactivation [37]. The induction of NF-κB target genes, such as *COX2*, can also lead to Nrf2 suppression [38]. Thus, anti-inflammatory agents, which suppress NF-κB signaling, may activate Nrf2 at the same time. Nrf2 also reverts the cellular damage of inflammation-exposed cells, thereby indirectly preventing sustained NF-κB activation [36].

Thus, it is possible that lichen secondary metabolites, particularly physodic and salazinic acids, may act similarly as anti-inflammatory agents by suppressing NF-κB and activating Nrf2 signaling.

NF-κB is functionally related to many other transcription factors, particularly the STAT family. NF-κB and STAT3 cooperatively regulate the expression of many proteins, especially anti-apoptotic and cell-cycle control proteins [12]. The activation of STAT3 is related to its phosphorylation and translocation to the cell nucleus.

Lichen metabolites, especially physodic acid and salazinic acid in DLD-1 cells, inhibited DNA binding and decreased p-STAT3 protein levels in the cell nucleus, which resulted in decreased expression of the anti-apoptotic protein Bcl-xl. A constitutively increased level of p-STAT3 was found in colorectal adenocarcinomas as opposed to STAT3 levels in the normal intestinal mucosa [39]. Modulation of the p-STAT3 level and its consequences under the influence of lichen metabolites is therefore definitely beneficial, as is the reduction of *Bcl-xl* expression, which can lead to increased apoptosis.

In HCT116 cells, a decreased level of p-STAT3 protein was found in the nuclear fraction, indicating its inhibition by physodic acid. The expression of target genes such as *Bcl-xl* was also decreased under the influence of physodic and salazinic acid, but only at the mRNA level, and it was not reflected in the decreased level of the protein encoded by this gene.

In summary, the results of our present study confirmed our earlier observations that lichen-derived compounds have the ability to modulate signaling pathway networks. While caperatic acid affected Wnt/β-catenin to the greatest extent, salazinic acid was the most potent modulator of the Nrf2, NF-κB and STAT3 pathways. On the other hand, physodic acid seemed to affect all the investigated pathways. Further studies are required to better explain the mechanism and significance of these observations.

## 4. Materials and Methods

### 4.1. Chemicals

Lichen components—Cap, Atra, Leca, Squam, Phys and Salaz—were isolated from the lichen specimens *Plasmatica glauca*, *Hypocenomyce scalaris, Cladonia uncialis*, *Hypogymnia physodes* and *Parmelia sulcata* as described previously [10].

Dimethyl sulfoxide (DMSO), antibiotics solution (10^4^ U penicillin, 10 mg streptomycin, 25 μg amphotericin B), fetal bovine serum (FBS), Dulbecco’s Modified Eagle’s Medium (DMEM) and Tris were obtained from Sigma-Aldrich, St. Louis, MO, USA. Human colorectal (HCT116 and DLD-1) cancer cells were obtained from the European Collection of Authenticated Cell Culture (Cell Lines Service, Eppelheim, Germany).

### 4.2. Cell Culture and Treatment

The HCT116 and DLD-1 cells were maintained in DMEM containing 10% FBS (EURx, Poland) and 1% antibiotics (Sigma-Aldrich, USA) and propagated in a humidified atmosphere with 5% CO_2_ at 37 °C. To assess the effect of lichen components on the measured parameters, 2 × 10^6^ cells were seeded per 100 mm ø culture dish. After 24 h of initial incubation, the cells were treated with 25 or 50 µM of lichen components or 0.1% of the vehicle control. The incubation was continued for the subsequent 48 h and the cells were harvested. The concentration of the studied compounds was selected based on the previously published cell viability data [10].

### 4.3. Cell Fractionation and Preparation of RNA

The cytosol and nuclear extracts from HCT116 and DLD-1 cells were prepared using a Nuclear/Cytosol Fractionation Kit (BioVision, Milpitas, CA, USA) according to the manufacturer’s protocol.

Extraction of total RNA from the cells was performed using a Universal RNA Purification Kit (EURx, Poland) according to the manufacturer’s instruction.

### 4.4. Western Blot

For the determination of the level of Nrf2, phosphorylated Nrf2 (p-Nrf2), SOD, GSTP, CAT, GPx, NF-κB subunits p50 and p65, COX-2, iNOS, STAT3, phosphorylated STAT3 (p-STAT3) and Bcl-xl proteins, the immunoblot assay was used. Protein content in the samples was determined by the Lowry method (1951). Nuclear (Nrf2, p-Nrf2, NF-κB p50, NF-κB p65, STAT3 and p-STAT3) or cytosolic proteins (Nrf2, GSTP, SOD, CAT, GPx, NF-κB subunits p50 and p65, COX-2, iNOS, STAT3 and Bcl-xl) (50–100 µg) were separated on 12%, 10% or 7.5% SDS-PAGE slab gels and proteins were transferred to nitrocellulose membranes. After blocking for 2 h with 10% skimmed milk, proteins were probed with primary antibodies against Nrf2 (sc–13032), p-Nrf2 (ab180844), SOD (sc–8637), GSTP (ab53943), CAT (sc–34281), GPx sc–30147), NF-κB p50 (sc–114), NF-κB p65 (sc–7151), COX-2 (sc–376861), iNOS (sc–651), STAT3 (sc–483), p-STAT3 (sc–7993), Bcl-xl (ab77571), β-actin (sc–7210) and lamin (sc–20680). The β-actin and lamin proteins were used as loading controls. As the secondary antibodies in the staining reaction, alkaline phosphatase (AP)-labeled anti-goat IgG, anti-mouse IgG or anti-rabbit IgG (Bio-Rad Laboratories, Hercules, CA, USA) and horseradish peroxidase (HRP)-conjugated anti-mouse IgG (Boster Bio, Pleasanton, CA, USA) were used. Bands were visualized with a BioRad AP Conjugate Substrate Kit NBT/BCIP or the chemiluminescent HRP-substrate Clarity ECL Kits (Bio-Rad Laboratories, Hercules, CA, USA). The amount of the immunoreactive product in each lane was determined using the ChemiDoc^TM^ Touch Imaging System (Bio-Rad Laboratories, Hercules, CA, USA). Protein levels are expressed in relative band blackness values (RQ).

### 4.5. ELISA Assay

Nrf2, NF-κB p50, NF-κB p65 and STAT3 activation were assessed by enzymatic immunoassay (Transcription Factor ELISA Assay Kit, Active Motif, La Hulpe, Belgium) according to the manufacturer’s instructions. Activated Nrf2 was evaluated based on the amount of Nrf2 contained in the DNA-binding complex to the ARE sequence. The oligonucleotides containing the ARE consensus-binding site (5′-GTCACAGTGACTCAGCAGAATCTG-3′) for Nrf2 were immobilized on microplates as bait. Activated NF-κB was measured as the amounts of p65 and p50 subunits held in the DNA-binding complex. The oligonucleotides containing (5′-GGGACTTTCC-3′) a consensus site for NF-κB were immobilized on microplates as bait. Activated STAT3 was measured as the amount of STAT3 contained in the DNA-binding complex. The oligonucleotides containing (5′-TTCCCGGAA-3′), a consensus site for STAT3, were immobilized on microplates as bait. Nuclear fractions were incubated with oligonucleotides for 1 h. Then, wells were washed and DNA-bound subunits were detected by the specific primary antibody and a secondary antibody conjugated with HRP. This allowed the colorimetric readout of the conjugate at 450 nm.

### 4.6. Quantitative Real-Time PCR (R-T PCR)

Revert-Aid First Strand cDNA Synthesis (Fermentas, Burlington, ON, Canada) was used for reverse transcription of RNA. For R-T PCR analyses, the Maxima SYBR Green Kit (Fermentas, Burlington, ON, Canada) and the LightCycler 96 (Roche, Mannheim, Germany) thermal cycler were used. The protocol started with 5 min enzyme activation at 95 °C, followed by 40 cycles of 95 °C for 15 s, 56 °C for 20 s, 72 °C for 40 s and final elongation at 72 °C for 5 min. Melting curve analysis was used for the verification of single-product amplification. The estimation of the expression of *TBP* (*TATA box binding protein*) and *PBGD* (*porphobilinogen deaminase*) was used for data normalization. The Pfaffl relative method [40] was used for fold-change quantification. The starters were designed using the Beacon Designer software. In addition, BLAST searches were performed to minimize non-specific binding. Consequently, only primer pairs generating amplicons spanning over introns were selected. All the oligonucleotides were ordered from the Institute of Biochemistry and Biophysics in Poland. Primer sequences are listed in Table 1.

### 4.7. Statistical Analysis

Statistical calculations were performed using the GraphPad Instat program (GraphPad Software, San Diego, CA, USA); the level of significance of changes was assumed as *p* ≤ 0.05. The differences between the mean values were assessed using Student’s *t*-test.

## Figures and Tables

**Table 1:** The sequences of primers used in the real-time PCR reactions.

Gene	Primer F (5′→3′)	Primer R (5′→3′)
*Nrf2*	ATTGCTACTAATCAGGCTCAG	GTTTGGCTTCTGGACTTGG
*SOD*	CGACAGAAGGAAAGTAATG	TGGATAGAGGATTAAAGTGAGG
*GSTP*	GCAAATACATCTCCCTCATC	AGGTTGTAGTCAGCGAAG
*CAT*	TGGACAAGTACAATGCTGAG	TTACACGGATGAACGCTAAG
*GPx*	CAACCAGTTTGGGCATCAG	TTCACCTCGCACTTCTCG
*NF-κB p50*	ATCATCCACCTTCATTCTCAA	AATCCTCCACCACATCTTCC
*NF-κB p65*	CGCCTGTCCTTTCTCATC	ACCTCAATGTCCTCTTTCTG
*COX-2*	CGCCTGTCCTTTCTCATC	CAGCCCGTTGGTGAAAGC
*iNOS*	AGGAGATGCTGAACTACG	GGATGGTGACTCTGACTC
*STAT3*	GCTTCTCCTTCTGGGTCTG	AGGCTTAGTGCTCAAGATGG
*Bcl-xl*	AAGCGTAGACAAGGAGATGC	CAGCGGTTGAAGCGTTCC
*PBGD*	TCAGATAGCATACAAGAGACC	TGGAATGTTACGAGCAGTG
*TBP*	GGCACCACTCCACTGTATC	GGGATTATATTCGGCGTTTCG

## Data Availability

The data are contained within this article.

## Sample Availability

Samples of the compounds (examined depsides, depsidones and caperatic acid) are available from the authors.
